# Time-Dependent Growth of Silica Shells on CdTe Quantum Dots

**DOI:** 10.3390/nano8060439

**Published:** 2018-06-16

**Authors:** Pavlína Modlitbová, Karel Klepárník, Zdeněk Farka, Pavel Pořízka, Petr Skládal, Karel Novotný, Jozef Kaiser

**Affiliations:** 1 Central European Institute of Technology (CEITEC) Brno University of Technology, Technická 3058/10, 61600 Brno, Czech Republic; pavel.porizka@ceitec.vutbr.cz (P.P.); jozef.kaiser@ceitec.vutbr.cz (J.K.); 2 Institute of Analytical Chemistry, Academy of Sciences of the Czech Republic, Veveří 97, 60200 Brno, Czech Republic; klep@iach.cz; 3 Central European Institute of Technology (CEITEC) Masaryk University, Kamenice 5, 62500 Brno, Czech Republic; farka@mail.muni.cz (Z.F.); skladal@sci.muni.cz (P.S.); codl@sci.muni.cz (K.N.)

**Keywords:** quantum dots, nanoparticles, photoluminescence spectra, dynamic light scattering, scanning electron microscopy

## Abstract

The purpose of this study is to investigate the time dependent growth of silica shells on CdTe quantum dots to get their optimum thicknesses for practical applications. The core/shell structured silica-coated CdTe quantum dots (CdTe/SiO_2_ QDs) were synthesized by the Ströber process, which used CdTe QDs co-stabilized by mercaptopropionic acid. The coating procedure used silane primer (3-mercaptopropyltrimethoxysilane) in order to make the quantum dots (QDs) surface vitreophilic. The total size of QDs was dependent on both the time of silica shell growth in the presence of sodium silicate, and on the presence of ethanol during this growth. The size of particles was monitored during the first 72 h using two principally different methods: Dynamic Light Scattering (DLS), and Scanning Electron Microscopy (SEM). The data obtained by both methods were compared and reasons for differences discussed. Without ethanol precipitation, the silica shell thickness grew slowly and increased the nanoparticle total size from approximately 23 nm up to almost 30 nm (DLS data), and up to almost 60 nm (SEM data) in three days. During the same time period but in the presence of ethanol, the size of CdTe/SiO_2_ QDs increased more significantly: up to 115 nm (DLS data) and up to 83 nm (SEM data). The variances occurring between silica shell thicknesses caused by different methods of silica growth, as well as by different evaluation methods, were discussed.

## 1. Introduction

Quantum dots (QDs) are fluorescent semiconductor nanocrystals which are being extensively developed because of their unique size-dependent optical and photophysical properties [1]. These properties have made QDs ideal for applications in mainstream market products [2], but most importantly, in science as labels for tagging and imaging in biological systems [3]. Various conjugation techniques of QDs, such as luminophors with antibodies as selectors, have been developed for bioanalytical applications [4,5]. The number of organic ligands attached to QD surfaces can be determined by various methods [6]. Nevertheless, the following problems remain when using QDs as fluorescent biological labels: (i) strong dependence of the fluorescence on surface states [7], (ii) cytotoxicity as a result of the release of heavy metal ions [8], (iii) chemical and colloidal instabilities of QDs in harsh environments, as well as (iv) toxicity for non-target organisms in the environment [9,10]. An effective solution for overcoming these drawbacks is the encapsulation of QDs by several types of shells. The most commonly used are ZnS [11], various polymers [12], and silicates [13,14].

Various nanoparticles (NPs) covered with an amorphous silica shell have shown a great success in impeding the leakage of heavy metal ions into the environment, suppressing surface defects caused by oxygen present in the surrounding media, enhancing the chemical stability of the NPs, and in supplying the silica shell with numerous functional groups, allowing for reaction control in bioconjugation protocols [13,15]. In the case of analytical and biological applications, smaller particle size is generally preferred due to the size compatibility of NPs with biomolecules [16], and higher biocompatibility [17]. In contrast, rather thick shells are appropriate mainly for specific purposes. Their application as active photonic crystals is very promising [18]. To enhance QDs’ stability, silica shells of 1–5 nm thickness [19] showed great stability of QDs for months in buffer solutions.

Silanization can be realized by two different approaches. The first is based on the reverse (water-in-oil) micro-emulsion method. Here, the original hydrophobic ligands are first replaced by hydrolyzed tetraethyl orthosilicate (TEOS), then the hydrophobic QDs enter the hydrophilic interior of the micelles where silica growth takes place. With respect to hydrophilic QDs, it is demonstrated that electrostatic repulsion is responsible for holding the core of QDs inside the resultant silica particles [13]. This micro-emulsion method commonly yields QDs with diameter sizes in the range of 30 to 150 nm [13,14]. The second approach is based on the Ströber process. Pre-coating by silane primers such as 3-mercaptopropyltrimethoxysilane (MPS) or 3-aminopropyltrimethoxysilane (APS) is typically required in order to make the QDs surface vitreophilic, which is a prerequisite for the QDs to act as nucleation sites for silica encapsulation [15]. The diameter of the final silica particles can be tuned from approximately 30 nm to 1 µm by means of seeded growth, e.g., QDs concentrations [18]. The smallest silanized QDs were prepared in the range from 4 to 16 nm [15]. Large silica spheres with size from 100 nm up to 700 nm could be prepared as well [20]. 

One of the first documented growths of silica shell onto QDs (CdS) was described as a procedure consisting of a slow deposition of a thin silica shell from a silicate solution followed by transfer into ethanol solution to grow thicker shells [21]. The MPS was used as a silane coupling agent as well, since it contains a mercapto group, directly bound to surface Cd atoms, conveniently leaving the silane groups pointing towards the solution from where the silicate ions approach the particle surface. These silicate ions build up a first silica shell, permitting transfer into ethanol without particle coagulation. The modification of ethanol transfer by the simple addition of pure ethanol into the silicate solution is called “ethanol precipitation” [15]. The addition of ethanol has two main goals: (i) decreasing of the silicate solubility (it serves to precipitate out all silicate moieties as monomers and/or oligomers still present in the solution), and (ii) the maximum silicate deposition on the cores achieved, while the spontaneous nucleation of silica particles is minimized [21].

In our opinion, the previous studies provided information about the silanization process in general, but they did not analyze the course of the silanization in enough detail [15,17]. Such an investigation is valuable with respect to the promising and extensive field of applications of silanized QDs, especially as non-toxic labels for tagging and imaging in biological systems. Thus, the objective of our paper is an inspection of the growth of silica shells on CdTe QDs nanoparticles in time. We compare the growth rate of amorphous silica shells onto as-synthesized water-soluble cadmium telluride QDs capped by 2-mercaptopropionic acid (CdTe MPA QDs) under various conditions. The main goal was to prepare silica-coated CdTe QDs (CdTe/SiO_2_ QDs) while observing and comparing the growth of the shell during ethanolic and non-ethanolic precipitation. In this study, the silanization is straightforward, and it is based on the ligand exchange of mercaptopropionic acid (MPA) with MPS molecules. The sizes of bare CdTe QDs and growth of silica shells in time were investigated in detail by several spectroscopic and microscopic techniques: Dynamic Light Scattering (DLS), Transmission Electron Microscopy (TEM), Scanning Electron Microscopy (SEM), and UV-VIS spectrometry. The obtained results provide a realistic view of the reliability of current experimental methods of QDs coating. 

## 2. Materials and Methods 

### 2.1. Chemicals and Materials

Sodium borohydride, anhydrous cadmium chloride, mercaptopropionic acid, together with isopropanol, were purchased from Lachner (Neratovice, Czech Republic). Sodium tellurite, sodium citrate tribasic dihydrate, sodium hydroxide, 3-mercaptopropyltrimethoxysilane, ethanol absolute, and sodium silicate (27% SiO_2_ in 14% NaOH) were obtained from Sigma Aldrich (Steinheim, Germany). Dialysis tubes Membra-CelTM (14 kDa molecular weight cutoff, #1784.1) were purchased from Roth (Karlsruhe, Germany). All chemicals used were of analytical grade or of the highest available purity. Deionized water underwent a demineralization by reverse osmosis. It was further purified using a Millipore RG (Merck KGaA, Darmstadt, Germany), and used throughout all experiments.

### 2.2. Synthesis of CdTe Nanocrystals

Water-soluble CdTe quantum dots were prepared by a modified one-step reaction described in our previous work [22]. Briefly, 69 mg of cadmium chloride was dissolved in 70 mL of Milli-Q water. Then, 200 mg of sodium citrate and 194 µL of mercaptopropionic acid (MPA) were added. Subsequently, pH was adjusted to 10 by the addition of 1 M sodium hydroxide. Finally, 18 mg of sodium tellurite and 100 mg of sodium borohydride were added, creating a total volume of 85 mL. The solution was heated under continuous stirring in a round bottom flask under reflux at 95 °C for 5 h. After cooling to room temperature, the solution was precipitated with isopropanol, and then centrifuged at 14,100 *g* (Eppendrof MiniSpin Plus, Eppendorf, Germany) for 10 min. The precipitated QDs could be suspended in deionized water or stored in solid state. A part of the crude product was immediately used for the silanization process.

### 2.3. Synthesis of CdTe/SiO_2_ Nanocrystals

Silanization was based on the methodology reported by [15], with only a slight modification applied, as follows. A volume of 10 mL of a crude CdTe-MPA QDs solution was mixed with 1.5 mL of MPS for 2 h to allow for surface priming. Then, this solution was removed into dialysis tubing and dialyzed versus 2 L of Milli-Q water (pH 11; pH was adjusted by the addition of 1 M sodium hydroxide) for at least 1 h. To assure a complete surface coverage, an additional 1.5 mL of MPS solution was added, and dialysis was continued for an additional 2 h. Then, the solution was slightly mixed with 2 mL of sodium silicate, and several aliquots were taken during shell growth for a detailed characterization. These aliquots were taken at the following times: 1, 2, 3, 4, 24, 28, 44, 48, 68, and 72 h. The solution could be stored in liquid state, or QDs could be precipitated by isopropyl alcohol and then centrifuged at 14,100 *g* for 10 min. The supernatant was discarded and QDs were dried at 45 °C and stored in a solid state.

### 2.4. Ethanol Precipitations of CdTe/SiO_2_ Nanocrystals

Ethanol precipitation was performed to facilitate the formation of thicker silica shells during the CdTe/SiO_2_ preparation. A volume of 1.0 mL of pure ethanol was added 1 h after the silanization, followed by the addition of sodium silicate. This solution was slightly mixed and several aliquots were removed during shell growth for a detailed characterization at the following times: 0.2, 1, 2, 24, 28, 44, 48, 68, and 72 h. After 72 h, the precipitated silica-coated QDs were centrifuged at 12,000 *g* for 2 min, the supernatant was discarded, silicate QDs were dried at 45 °C, and both parts were stored.

### 2.5. Photophysical and Size Characterization

Nominal hydrodynamic particle diameter was determined by the DLS method. All DLS measurements were performed at the room temperature (25 ± 2 °C), with a Zetasizer Nano ZS (Malvern Instruments, Worcestershire, UK) equipped with a He-Ne laser (Worcestershire, UK) (λ = 633 nm) and a backscatter detector (Worcestershire, UK) at a fixed angle of 173°. The instrument recorded the intensity of autocorrelation function which was transformed into volume functions to obtain size information. The particle concentration for the DLS experiments was approximately 2.3 × 10^12^ NPs/mL. Emission spectra were measured with a spectrofluorometer FP-8500 (Jasco, Easton, Los Angeles, CA, USA). For Transmission electron microscopy, images were obtained with a FEI Tecnai F20 (FEI, Eindhoven, The Netherlands) operating at 200 kV. The QDs solution was deposited on a carbon-modified TEM target and vitrified using a FEI Vitrobot (Eindhoven, The Netherlands). The sample under liquid nitrogen was transferred into TEM, and individual images were collected with 16k CCD camera (FEI Eagle, Eindhoven, The Netherlands). The SEM pictures were taken with a MIRA 3 (Tescan, Brno, Czech Republic) at 10 kV with a magnification of 150,000 times. The TEM and SEM images were evaluated using ImageJ software (National Institutes of Health, Bethesda, MD, USA).

## 3. Results and Discussion

Silanized QDs exhibit many benefits; they could be notably used as precursors for the preparation of non-toxic labels for tagging and imaging in biological systems due to their easy functionalization with several biolinkers [15,23]. The increased stability of silanized QDs was investigated several times. As an example, the stability in tris-borate-ethylenediaminetetraacetate and phosphate-buffered saline buffers could be mentioned [15]. Mulvaney et al. found a silica shell to be a useful tool for minimizing of fluorescence quenching [24]. Great stability against photodegradation of silica-coated QDs was established [21,24]. This effect was connected to the small pore size of the silica shells, which made it very difficult for oxygen molecules to reach the particle surface. This situation was described for various chemical reactions performed on silica-coated metal (Au and Ag) NPs as well [25].

### 3.1. Photophysical Properties

**Figure 1:**
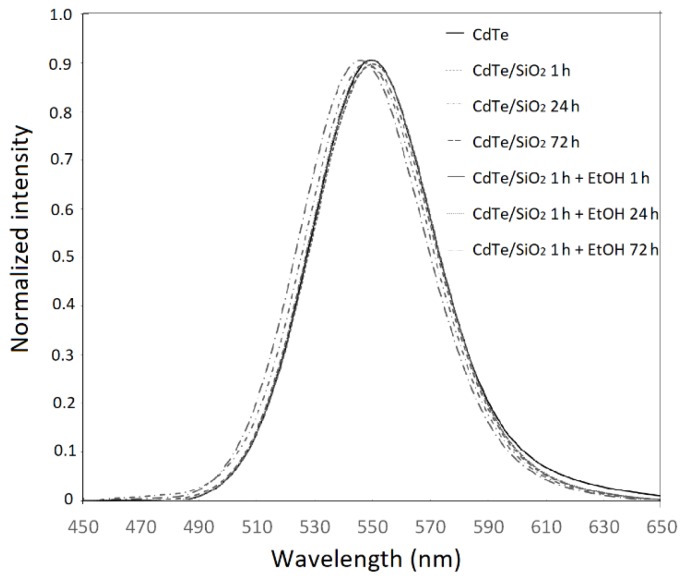
The emission spectra of as-synthesized CdTe quantum dots(QDs) and the silanized aliquots of CdTe QDs with and without presence of ethanol in specific times (1, 24, and 72 h).

The emission spectrum of as-synthesized CdTe QDs is shown in Figure 1. The spectra of the CdTe/SiO_2_ QDs correspond to the particles taken out of the reaction vessel during the growth phase with and without ethanol at 1, 24, and 72 h. The spectra show that, during the growth of silica shell, only small changes of maxima wavelength and intensity appeared. Thus, the fluorescence wavelength of our CdTe/SiO_2_ QDs is nearly independent of the silanization time. This is a confirmation of the fact that silica coatings do not affect the luminescence of QDs particles, and the process of coating does not affect a size of CdTe core. A similar behavior was observed by Jing et al. [13]. In contrast, Wolcott et al. [15] found a shift of a maximum of between 555 nm (unmodified CdTe QDs) to 580 nm after the silica shell was formed. This fact was explained as a result of passivation of the surface by an exchange of ligands from TGA (thioglycolic acid) to MPS, and by the decrease in charged species on the surface [15].

### 3.2. Size Characterization

**Figure 2:**
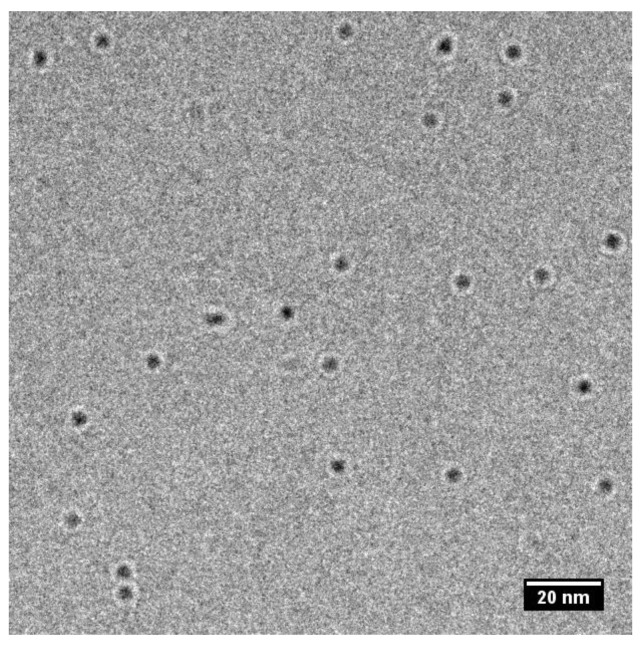
Transmission Electron Microscopy (TEM) picture of CdTe QDs.

Average particle sizes of bare CdTe QDs in aqueous solutions were measured by two principally different methods, TEM (Figure 2) and DLS. The sizes obtained with both methods are 3.8 ± 0.3 nm and 3.5 ± 0.2 nm, so they are comparable (Table 1). Moreover, both results are comparable with the size 3.1 nm evaluated from the wavelength of maxima of luminescence spectra [4]. It should be emphasized that the QDs are present as individual particles without any aggregation.

**Figure 3:**
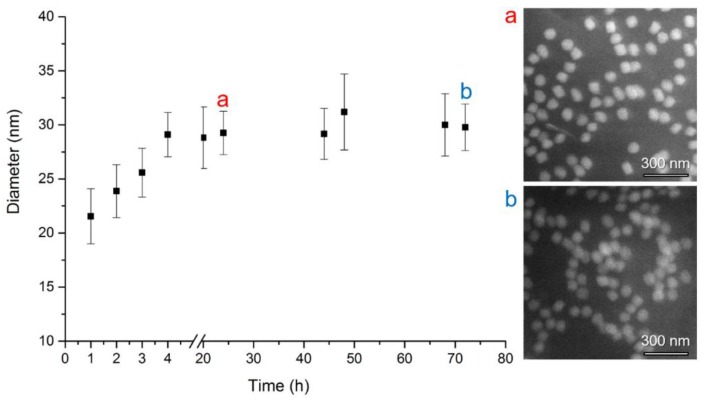
Dependence of CdTe/SiO_2_ QDs average hydrodynamic particle diameter (nm) on time (hours) of silica shell growth. The number of Dynamic Light Scattering (DLS) measurements per one exposure time was 5, error bars correspond to standard deviations. Scanning Electron Microscopy (SEM) photograh (**a**) after 24 h growth (diameter 53.7 ± 4.2 nm) and (**b**) after 72 h growth (diameter 59.7 ± 4.0 nm).

In our study, we monitored an increase of particle diameter with the time of silica shell growth. The results of DLS measurements are demonstrated in Figure 3 and summarized in Table 1. As shown here, without any ethanol precipitation, the diameter size of 23 nm grows up to 30 nm in 4 h, and does not change substantially after 72 h (DLS data). The obtained DLS characteristics were complemented by SEM micrographs, as presented in Figure 3 by inset (a) and (b) and in Table 1. A similar size of both NPs was observed after 24 h (53.7 ± 4.2 nm) and 72 h (59.7 ± 4.0 nm) by SEM method as well. 

**Figure 4:**
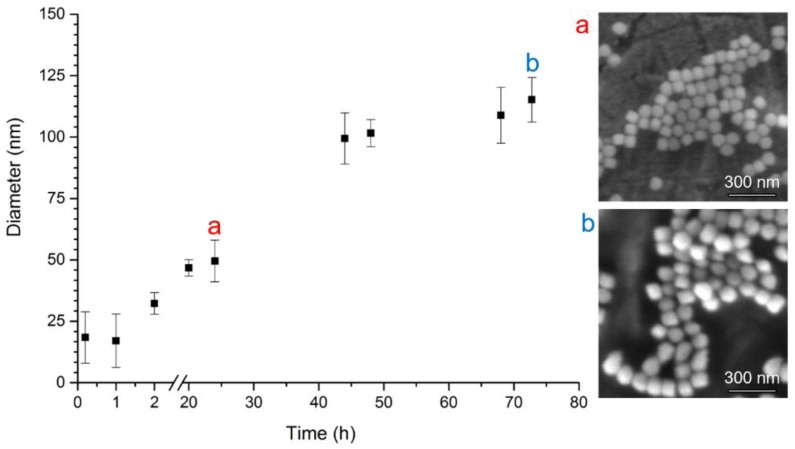
Dependence of CdTe/SiO_2_ QDs average hydrodynamic particle diameter (nm) on time (hours) of silica shell growth in ethanol presence. The number of DLS measurements per one exposure time was 5; error bars correspond to standard deviations. SEM photograh (**a**) after 24 h growth (diameter 62.3 ± 7.5 nm) and (**b**) after 72 h growth (diameter 83.1 ± 6.9 nm).

A more pronounced increase of silica shell thickness caused by ethanol precipitation is demonstrated by the DLS measurements shown in Figure 4. Every 24 h, the diameter increases significantly., from approximately 25 nm at the beginning to 50 nm after 24 h, 100 nm after 48 h, and 115 nm after 72 h of the synthesis. The continuous trend in NP growth was confirmed by SEM data as well. The size of the particles is evaluated to be 62.3 ± 7.5 nm after 24 h, and increased up to 83.1 ± 6.9 nm after 72 h of the synthesis. Thus, the ethanol precipitation is a method of choice when thick SiO_2_ coatings or large particles of predefined sizes are to be formed.

Although the trend of increasing size with the time of synthesis determined by SEM and DLS is clear, it is noticeable that both methods do not provide exactly the same values. In our opinion, this is caused by the irregular shapes of silica coated NPs. The particle shape, as well as the particle size distribution, is important in obtaining a meaningful size diameter. This was already mentioned in several studies [26,27,28,29]. The difference in sizes of spherical bare QDs evaluated by TEM and DLS is in the range of about 8%, while the difference in sizes of coated particles evaluated by SEM and DLS is at least around 20%.

A similar situation has been well documented by others [26,27]. In the first [27] study, they presented different diameters of Ag and Au NPs obtained by SEM and DLS methods as number, volume, and intensity distributions. For Au NPs, the size was established to be 13 nm by SEM, while DLS provided the sizes 22 ± 7, 52 ± 23 and 30 ± 13 nm, as the maxima of number, volume, and intensity distributions, respectively. The most commonly used size from the DLS method is a value obtained from number data distribution, in this case 22 ± 7 nm, which is almost two times bigger than the value from SEM. This trend was very similar to our data for CdTe/SiO_2_ QDs after 72 h of ethanol precipitation, as shown in Table 1 (SEM—83.1 ± 6.9, DLS—115.1 ± 13.7 nm). In contrast, the opposite trend was observed for Ag NPs, where SEM diameter size was 70 ± 19 nm, while DLS provided the sizes 63 ± 21, 124 ± 50, and 94 ± 47 nm, as the maxima of number, volume, and intensity distributions, respectively. Again, the most commonly used size diameter is 63 ± 21 nm, which is distinctly lower (10% variance) than the value obtained from SEM. This situation occurred in our investigation as well: CdTe/SiO_2_ QDs without ethanol precipitation and also for CdTe/SiO_2_ QDs after 24 h of ethanol precipitation, as summarized in Table 1.

Our data could be also compared with results of poly(butyl cyanoacrylate) NPs (PBCA NPs) [26]. In this study, the size diameter of PBCA NPs obtained from SEM was 167 ± 16 nm (determined from 156 particles), with a narrow size distribution and DLS, has shown that the mean hydrodynamic particle diameter is 192 ± 7 nm; this depends on the concertation of measured NPs solution. Moreover, the recognizable variations among SEM and DLS results appeared frequently. Typically, the diameter size obtained from DLS is bigger than from SEM (TEM) [26]. This difference is explained by the changes in particle properties during sample preparation. For instance, drying on the air and freeze-drying causes shrinking of the particles [26]. An opposite situation, based on our results together with the literature research, could be caused by non-spherical shape of NPs, as well as non-narrow particle size distribution [27,28,29].

In the DLS system, the sizes of particles are calculated from the rate of scattered light intensity fluctuations controlled by Brownian motion, and thus, by the Stokes-Einstein equation. In fact, the instrument measures the degree of correlation between two signals of many particles over a period of time. Thus, in general, DLS measurements require a larger number of particles (several orders of magnitude greater) compared to SEM, and provide much better statistics. In contrast, the SEM method gives a detailed shape and morphological information. Thus, the particle diameters determined by SEM have to be considered as the lower limits of particle size. The main advantage of the DLS method is the short time required to perform the measurements. However, even the DLS method has several pitfalls, in particular with respect to the influence of dust particles or small amounts of large aggregates in addition to a main component of distinctly smaller size, and missing necessary information about the particle shape. Furthermore, the DLS provides information about the diameter of a sphere which is hydrodynamically equivalent to NPs, about different shapes of nanoparticles, and any other covalently or noncovalently attached molecules or solvents that move with the particle affect evaluations. However, TEM or SEM themselves provide only a set of actual sizes of nanoparticles which must be processed statistically. Therefore, it has to be mentioned that none of the used analytical methods for a particle size evaluation offers a universal solution. Thus, several complementary methods should be used to characterize sample size, shape, and morphology. Nevertheless, the DSL and SEM results reliably confirm the same trend in silica-shell growth.

## 4. Conclusions

In summary, we have presented a method to coat luminescent QDs with silica shells based on the Ströber process. It is demonstrated that silica coated QDs of various sizes could be prepared by the manipulation of physicochemical experimental conditions. The diameters of the final CdTe/SiO_2_ QDs can be synthesized from approximately 20 nm up to 115 nm by means of the time of growth in the presence of sodium silicate and by optional ethanol precipitation. During three days of well-documented silica shell growth without ethanol precipitation, we observed a moderate increase of the shell thickness, to give total particle sizes between approximately 23 to almost 30 nm (DLS data) and up to almost 60 nm (SEM data). During the same period, but in the presence of ethanol, the size of CdTe/SiO_2_ QDs increased more significantly: up to 115 nm (DLS data) and up to 83 nm (SEM data). The method based on ethanol precipitation provides time dependent growth for more than 50 h, whereas in the method without ethanol, the maximum particle size is reached after approximately 4 h.

To the best of our knowledge, there are no detailed studies showing the growth of silica shell thickness in the first hours of synthesis. Our results show that, in the case of preparation of CdTe/SiO_2_ QDs of a sizes less than 20 nm, it is necessary to restrict the time of growth in the presence of sodium silicate to only a few hours. Moreover, ethanol precipitation is not a convenient strategy in this case. The opposite is true for the preparation of large particles with a thick SiO_2_ coatings, where ethanol precipitation is a prerequisite of an effective synthesis. 

In addition, our results show that none of the widely used analytical methods for particle size evaluation is a universal solution, and several complementary methods should be used to obtain a reliable characterization of size, shape, and morphology.

## Figures and Tables

**Table 1:** Quantum dots (QDs) average hydrodynamic particle diameter (nm) obtained by Dynamic Light Scattering (DLS), and average particle diameter (nm) obtained by Scanning Electron Microscopy (SEM) and Transmission Electron Microscopy (TEM) measurements. Result obtained by TEM is marked by *.

QDs	Time of Silica Shell Growth (h)	DLS (nm)	SEM/TEM * (nm)
CdTe	/	3.5 ± 0.2	3.8 ± 0.3 *
CdTe/SiO_2_	24	28.8 ± 2.2	53.7 ± 4.2
CdTe/SiO_2_	72	29.2 ± 2.2	59.7 ± 4.0
CdTe/SiO_2_ + EtOH	24	50.1 ± 9.3	62.3 ± 7.5
CdTe/SiO_2_ + EtOH	72	115.1 ± 13.7	83.1 ± 6.9
